# Human IgM antibody rHIgM22 promotes phagocytic clearance of myelin debris by microglia

**DOI:** 10.1038/s41598-018-27559-y

**Published:** 2018-06-20

**Authors:** Yana Zorina, Jason Stricker, Anthony O. Caggiano, Donald C. Button

**Affiliations:** 1 Gene Editing and Screening Core Facility, Memorial Sloan Kettering Cancer Center, New York, NY USA; 2 Translational Medicine, Celgene Corporation, Summit, NJ USA; 3 Research and Development, Acorda Therapeutics Inc., Ardsley, NY USA; 4 PharmAble, San Francisco, CA, USA

## Abstract

In multiple sclerosis (MS), demyelinated CNS lesions fail to sufficiently remyelinate, despite the presence of oligodendrocyte precursor cells (OPCs) capable of differentiating into mature oligodendrocytes. MS lesions contain damaged myelin debris that can inhibit OPC maturation and hinder repair. rHIgM22 is an experimental human recombinant IgM antibody that promotes remyelination in animal models and is being examined in patients with MS. rHIgM22 binds to CNS myelin and partially rescues OPC process outgrowth on myelin. Since rHIgM22 does not affect OPC process outgrowth *in vitro* on permissive substrate, we examined the possibility that it acts by enhancing phagocytic clearance of myelin debris by microglia. In this study, we tested if rHIgM22 binding could tag myelin for microglial phagocytosis. A mouse microglial cell line and primary rat microglia were treated with myelin and rHIgM22 and assayed for myelin phagocytosis. We found that: 1) rHIgM22 stimulates myelin phagocytosis in a dose-dependent manner; 2) rHIgM22-mediated myelin phagocytosis requires actin polymerization; and 3) rHIgM22-stimulation of myelin phagocytosis requires activity of rHIgM22 Fc domain and activation of Complement Receptor 3. Since myelin inhibits OPC differentiation, stimulation of phagocytic clearance of damaged myelin may be an important means by which rHIgM22 promotes remyelination.

## Introduction

Activation of the immune system is believed to be one of the main causes of many neurodegenerative disorders. In multiple sclerosis (MS), activated immune cells specifically attack myelin sheaths that insulate axons, leading to myelin degradation and ultimately neurodegeneration. While activation of the immune system and generation of autoantibodies has traditionally been seen as one of the hallmarks of MS pathology, natural IgM antibodies have also been shown to have restorative and beneficial functions in the body^1^. rHIgM22 is a recombinant version of a naturally occurring, human IgM that has been shown to promote remyelination in the Theiler’s virus infection-induced^2^ and curpizone-mediated^3^ animal models of MS. A recently completed Phase 1 clinical trial demonstrated that single infusions of rHIgM22 were well tolerated by patients with clinically stable MS^4^. While the patient cohort was not large enough to detect significant changes in clinical outcomes, Patient Global Impression of Change showed a positive trend in patients treated with rHIgM22.

Most preclinical studies of rHIgM22 have been performed with *in vivo* systems, where identifying the specific cellular activity of rHIgM22 is challenging. While OPCs would appear to be good candidates for playing a central role in the remyelinating process(es) induced by rHIgM22, purified OPC *in vitro* cultures do not appear to respond to rHIgM22 treatment^5^. Instead, mixed glial cultures, which consist of astrocytes, OPCs, maturing oligodendrocytes (OLs) and microglia are required to detect cellular responses to rHIgM22 *in vitro*^5^. Under these conditions, OPCs show enhanced BrdU incorporation and Ki-67 expression after a 48-hour treatment. These findings suggest that other cell types may be required for rHIgM22 to exert its effect(s).

rHIgM22 was discovered by screening antibodies for binding to myelin followed by testing in the Theiler’s virus model^6^, but the specific antigen(s) with which it interacts has not been identified. Interestingly, rHIgM22 has been shown to bind directly to differentiated OLs^2,7^, but binding to immature OPCs has not been detected. Consistent with this, no responses to rHIgM22 have been detected in purified OPC cultures^5^. Since OPCs apparently do not respond to rHIgM22, and differentiated oligodendrocytes are unlikely to be sufficient for substantial remyelination, we sought to better understand how the ability of rHIgM22 to bind myelin plays a role in its effects on remyelination.

Central nervous system (CNS) myelin has been extensively studied in the context of acute CNS injury, where tissue damage leads to exposure of myelin-associated inhibitors that prevent axonal regeneration^8^. Similarly, MS lesions can result in exposure of damaged myelin debris, which over the last decade has been shown to exert inhibitory effects on OPC differentiation as well^9–13^. Given the ability of rHIgM22 to bind CNS myelin, we decided to explore if rHIgM22 could act as a classical IgM and aid in clearance of myelin debris, potentially allowing for disinhibition of OPC differentiation.

Natural IgMs can recognize apoptotic cells and enhance phagocytic clearance of cellular debris, thereby preventing unnecessary and potentially damaging inflammatory responses to host cell turnover^1,14^. Natural IgMs are poly-reactive and can bind to multiple endogenous antigens, which include proteins and lipids^1,15^. In turn, myelin consists of a complex mixture of a large number of proteins and lipids^16^, and rHIgM22 has been suggested to bind to a protein/lipid complex on myelin and OL surfaces^17^. Binding of IgM to myelin can recruit microglia, which recognize IgMs and act as the main phagocytic cells in the CNS^18,19^. Microglia have been reported to phagocytose (a) apoptotic cell debris under normal homeostatic conditions^19^; (b) plaque proteins in neurodegeneration^20,21^; (c) synapses in developmental synaptic pruning and Alzheimer’s disease^22,23^, and (d) myelin debris under demyelinating conditions^24^.

In this study, we tested if the ability of rHIgM22 to bind CNS myelin could enhance phagocytic clearance of myelin debris by microglia *in vitro*. Using a microglial cell line (BV-2), myelin phagocytosis was evaluated by examining the translocation of myelin to lysosomal compartments and biochemical detection of intracellular 2′,3′-cyclic-nucleotide 3′-phosphodiesterase (CNPase). CNPase is a myelin-associated enzyme, which is localized almost exclusively in myelinating cells - oligodendrocytes in the CNS and Schwann cells in the PNS^25^. CNPase is also one of the most abundant proteins in myelin^16^ and is commonly used as a marker of oligodendrocyte maturation^26^. Therefore, detection of CNPase in BV-2 cell lysates was used as an indicator of extracellular myelin uptake by BV-2 cells. We show that rHIgM22 can enhance microglial uptake and degradation of myelin. As would be expected for a phagocytic response, microglial uptake of myelin requires cytoskeletal rearrangements for the cells to be able to engulf the myelin. Furthermore, the process requires activity of the Fc portion of rHIgM22 for efficient presentation of rHIgM22-bound myelin to the BV-2 cells. In turn, we show that BV-2 cells recognize rHIgM22-opsonized myelin through complement receptor 3 (CR3), which has recently been shown to promote IgM-mediated phagocytosis^27^.

## Results

### rHIgM22 promotes microglial phagocytosis of myelin

**Figure 1:**
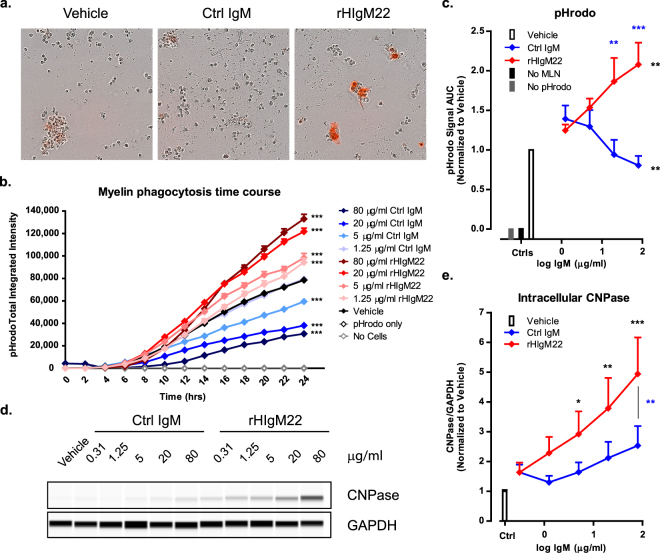
Stimulation of BV-2 cells with rHIgM22 promotes myelin phagocytosis. (**a,c**) BV-2 cells were serum starved and treated with pHrodo-labeled myelin and IgMs. pHrodo signal was monitored on IncuCyte ZOOM for 24 hours. (**a**) Representative images of BV-2 cells treated with Vehicle or IgMs (80 µg/mL) at 24 hours after treatment. (**b**) A representative time course of pHrodo signal quantification for 24 hours after treatment. Statistical differences were calculated using 2-way ANOVA followed by Bonferroni posttests. Black asterisks indicate significant difference compared to vehicle-treated cells at the corresponding time point (more detailed analysis is provided in Supplementary Table 1). (**c**) Quantification of pHrodo signal over 24 hours represented as area under the curve. The line graph shows the mean ± S.E.M. from 7 independent experiments. Statistical differences were calculated using 2-way ANOVA followed by Bonferroni posttests for rHIgM22 and isotype Ctrl IgM comparison (blue asterisks) and linear regression analysis for comparison to vehicle (black asterisks, details provided in Supplementary Fig. 5). (**d**) BV-2 cells were serum starved and treated with myelin and IgMs for 2 hours. The cells were lysed and analyzed for internalized CNPase by capillary immunodetection. Full blot is provided in Supplementary Fig. 6a. (**e**) The line graph shows mean ± S.E.M. of CNPase/GAPDH ratio from 7 independent experiments. Statistical differences were calculated using 2-way ANOVA followed by Bonferroni posttests. Black asterisks indicate significant difference compared to Vehicle treated cells. Blue asterisks indicate significant difference compared to cells treated with isotype Ctrl IgM at the corresponding concentration. (***p < 0.001; **p < 0.01; *p < 0.05).

To test if rHIgM22 can promote clearance of myelin debris, BV-2 cells were serum starved for two hours and treated with pHrodo-labeled myelin and rHIgM22 followed by monitoring pHrodo signal over the course of 24 hours **(**Fig. 1a,b**)**. Figure 1b shows a representative plot from a single experiment in which triplicate samples of each treatment condition were measured for total integrated intensity of pHrodo signal over the first 24 hours. Time course data were transformed into dose response curves by calculating area under the curve (AUC) of pHrodo total integrated intensity for each treatment, and averaged across experiments **(**Fig. 1c**)**. While all cells showed uptake of pHrodo-labeled myelin, treatment with rHIgM22 resulted in a ~2-fold increase of myelin uptake, whereas treatment with the isotype Ctrl IgM resulted in partial decrease of basal levels of myelin uptake over the course of 24 hours. Figures 1b and c show that response to Ctrl IgM was significantly lower than response to Vehicle. In order to confirm specificity of this response to myelin substrate, the same experiment was repeated using HEK293 cell membranes in place of myelin. While BV-2 cells internalized HEK293 membranes in response to all treatments, no difference was detected between vehicle, Ctrl IgM and rHIgM22-treated cells **(**Supplementary Fig. 1**)**.

To extend these observations made with pHrodo assays, we investigated rHIgM22 effects on BV-2 cell phagocytosis of myelin at early time points of treatment using a biochemical assay. BV-2 cells were treated with myelin and IgMs, and cell lysates were analyzed for the presence of CNPase by capillary electrophoresis immunoassay analysis (WES™). Figure 1d shows a representative capillary immunoblot of CNPase in BV-2 cells at 2 hours after treatment, and Fig. 1e shows the quantification of CNPase/GAPDH ratio averaged across experiments. Intracellular CNPase increased in a rHIgM22 dose-dependent manner, whereas CNPase levels did not change in response to Ctrl IgM treatment **(**Fig. 1d,e**)**. To verify that increases in CNPase levels indicated uptake of extracellular myelin, and not cellular synthesis of CNPase^28^, we analyzed cell lysates of BV-2 cells treated with rHIgM22 in the presence and absence of myelin. CNPase signal was detected only in cells treated with myelin, thereby making it a reliable marker of myelin phagocytosis **(**Supplementary Fig. 2**)**. To further validate that BV-2 cells phagocytose myelin in response to rHIgM22 treatment, we performed cell lysate fractionation at the 2-hour time point to confirm that CNPase signal can be attributed to internalized myelin. CNPase signal was detected only in cytosolic, but not plasma membrane fractions, indicating that non-specific myelin adsorption to cell surfaces was minimal and confirming that it can be used as a reliable measure of phagocytic uptake of myelin **(**Supplementary Fig. 3**)**.

**Figure 2:**
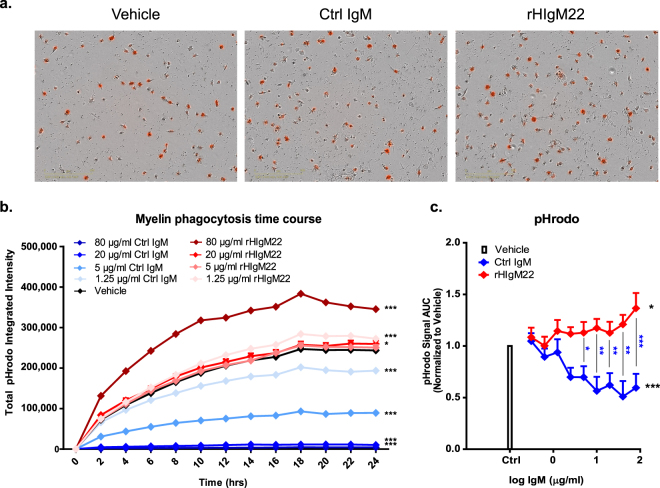
Stimulation of primary microglia with rHIgM22 promotes myelin phagocytosis. (**a**) Primary rat microglia were serum starved and treated with pHrodo-labeled myelin and IgMs. pHrodo signal was monitored on IncuCyte ZOOM for 24 hours. Representative images of cells treated with vehicle or IgMs (80 µg/mL) at 24 hours after treatment are shown. (**b**) A representative time course of pHrodo signal quantification for 24 hours after treatment. For clarity, curves for every other tested concentration are shown. Statistical differences were calculated using 2-way ANOVA followed by Bonferroni posttests. Black asterisks indicate significant difference compared to vehicle-treated cells at the corresponding time point (more detailed analysis is provided in Supplementary Table 2). (**c**) Quantification of pHrodo signal over 24 hours represented as area under the curve. The line graph shows the mean ± S.E.M. from 7 independent experiments. Statistical differences were calculated using 2-way ANOVA followed by Bonferroni posttests for rHIgM22 and isotype Ctrl IgM comparison (blue asterisks) and linear regression analysis for comparison to vehicle (black asterisks) (details provided in Supplementary Fig. 5). (***p < 0.001; **p < 0.01; *p < 0.05).

To confirm that the observed effects of rHIgM22 on myelin phagocytosis in BV-2 cells extend to primary microglial cells, rat microglia were purified and treated with pHrodo-labeled myelin and IgMs for 24 hours **(**Fig. 2a**)**. In primary microglia, the process of phagocytosis (pHrodo signal) appeared earlier (2 hours after treatment) **(**Fig. 2b**)** than in BV-2 cells that showed a lag of 8–10 hours to the initial response (Fig. 1b**)**. Similarly, in primary microglia the difference in response to rHIgM22 and Ctrl IgM was observed within 2–4 hours after treatment **(**Fig. 2b**)**, compared to a much slower divergence in BV-2 cells (>10 hours) **(**Fig. 1b**)**. Moreover, under all treatment conditions, higher total integrated intensity of pHrodo was observed in primary microglia than in BV-2 cells, suggesting that primary microglia are more efficient phagocytes. In the presence of higher basal activity of primary microglia, rHIgM22 treatment resulted in a weaker positive trend of myelin uptake, whereas the Ctrl IgM treatment resulted in greater inhibition of basal myelin uptake over 24 hours **(**Fig. 2c**)**. Due to the narrow dynamic range of response to rHIgM22 in primary microglia, the rest of this study was performed in BV-2 cells. In addition, in contrast to limited amounts of microglia that could be isolated from primary glial cultures, the BV-2 cell line allowed us to perform extensive biochemical studies of rHIgM22-mediated phagocytosis.

**Figure 3:**
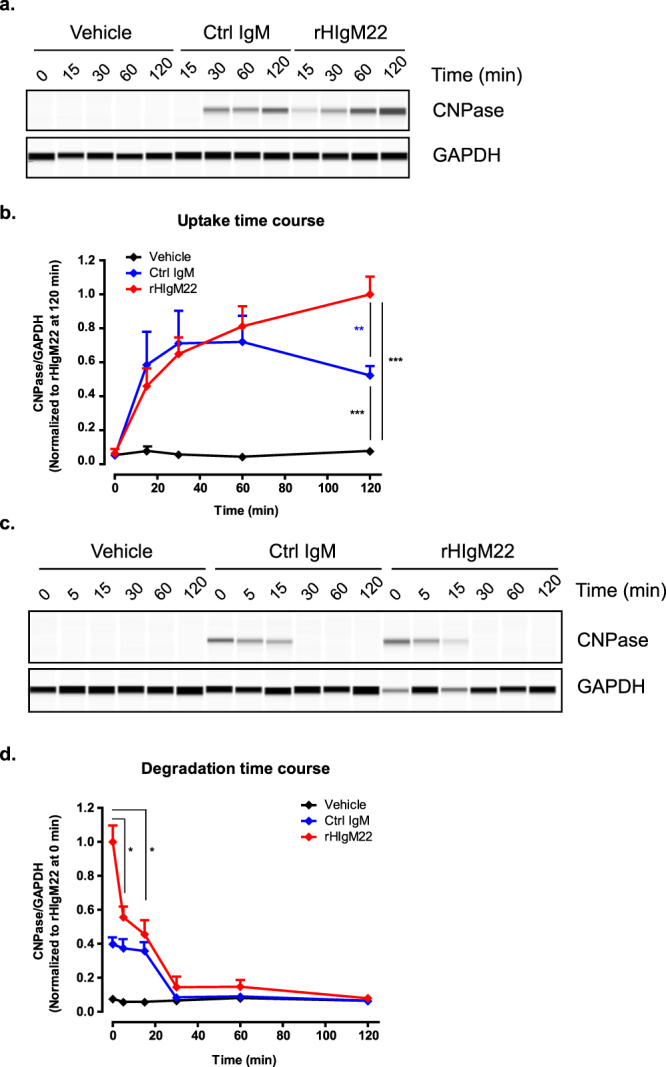
rHIgM22 promotes sustained myelin uptake and accelerates myelin degradation by BV-2 microglial cells. (**a,b**) For uptake time courses, BV-2 cells were serum starved, treated with IgMs (20 µg/mL) and lysed at indicated times. Lysates were analyzed by capillary immunoblot for CNPase. All CNPase/GAPDH ratio values were normalized to rHIgM22 treatment at 120 minutes as the maximal value. (**b**) The line graph shows the mean ± S.E.M. from 6 independent experiments. Statistical differences were calculated using t-test analysis (***p < 0.001; **p < 0.005). (**c,d**) For degradation time courses, BV-2 cells were serum starved, treated with IgMs (20 µg/mL) for 2 hours and switched to fresh media. Cells were collected at indicated time points after the media change to analyze remaining internalized myelin. (**d**) The line graph shows the mean ± S.E.M. from 3 independent experiments. To facilitate comparison to uptake time course data, all values were normalized to rHIgM22 treatment at 0 minutes, which corresponds to 120 minutes after addition of myelin and IgMs. Levels of remaining CNPase were compared to time point 0 by using t-test analysis (*p < 0.05). Full blots are provided in Supplementary Fig. 6b,c.

Phagocytosis has been reported to occur within 30–60 minutes of antigen presentation and be accelerated by addition of antigen-binding antibodies^21,29^. In BV-2 cells, we observed a difference in CNPase uptake at 2 hours **(**Fig. 1d,e**)**, which is earlier than the time point at which pHrodo signal levels began to diverge **(**Fig. 1b**)**. To compare effects of IgM antibodies on the rate of early myelin uptake, we performed CNPase analysis time course experiments over the first 2 hours of treatment, a time at which the pHrodo assay was not sensitive enough to detect a difference in signal. BV-2 cells were collected at multiple time points after addition of myelin and IgM treatment (20 µg/ml) and cell lysates were subjected to capillary immunoblot analysis for CNPase **(**Fig. 3a). Figure 3b shows the quantification of CNPase/GAPDH ratio. Internalized myelin levels were similar in rHIgM22 and Ctrl IgM-treated samples at early time points. Interestingly, only rHIgM22 treatment resulted in sustained myelin phagocytosis at 2 hours, as measured by biochemical analysis and indicated by continuous increase in levels of CNPase over time **(**Fig. 3a,b). Consistent with immunodetection analysis of lysates at 2 hours **(**Fig. 1d,e), treatment with Ctrl IgM resulted in increased levels of myelin uptake compared to vehicle, but lower than levels observed in rHIgM22-treated samples. From 60 to 120 minutes, the response to Ctrl IgM began to decline, potentially corresponding to lower pHrodo intensity level at later time points (as compared to vehicle).

Similarly, to test the degradation rates of myelin, we pre-incubated the cells with myelin and IgMs for 2 hours, performed a complete media change to wash away extracellular myelin, and collected cells at the indicated time points after the washout for capillary immunoblot analysis **(**Fig. 3c**)**. Intracellular CNPase levels were significantly decreased within the first 5 minutes after washout in the rHIgM22-treated cells, whereas in cells treated with Ctrl IgM myelin levels began to decrease after 30 minutes **(**Fig. 3c,d). These results suggest rHIgM22 promotes sustained myelin uptake and accelerates myelin degradation in microglial cells, whereas Ctrl IgM promotes myelin uptake only transiently and is not effective at increasing lysosomal degradation.

### rHIgM22-mediated phagocytosis of myelin requires actin polymerization

**Figure 4:**
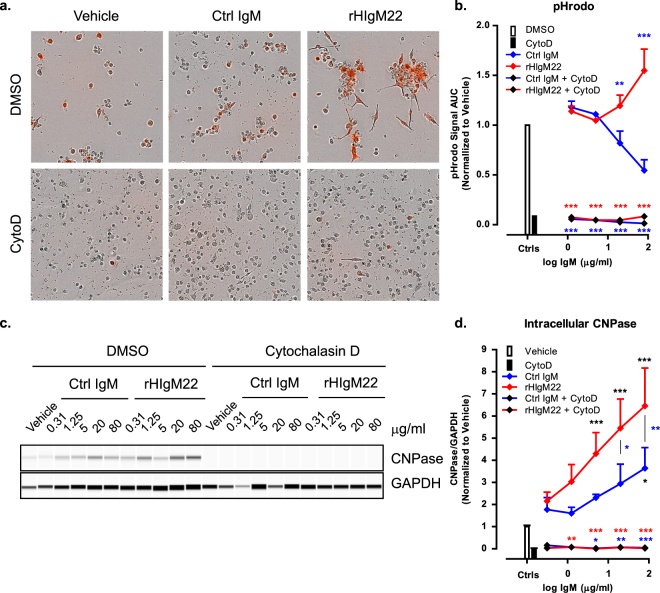
rHIgM22-mediated phagocytosis of myelin requires actin polymerization. (**a,b**) BV-2 cells were serum starved and pre-treated with Cytochalasin D for 30 minutes before application of pHrodo-labeled myelin and IgMs. pHrodo signal was monitored on IncuCyte ZOOM for 24 hours. (**a**) Representative images of BV-2 cells treated with vehicle or IgMs (80 µg/mL) in the presence or absence of Cytochalasin D at 24 hours after treatment. (**b**) Quantification of pHrodo signal over 24 hours represented as area under the curve. The line graph shows the mean ± S.E.M. from 3 independent experiments. Statistical differences were calculated using 2-way ANOVA followed by Bonferroni posttests for rHIgM22 and isotype Ctrl IgM comparison (blue asterisks) and linear regression analysis for comparison to vehicle (details provided in Supplementary Fig. 5). (**c**) BV-2 cells were serum starved, pre-treated with Cytochalasin D for 30 minutes and treated with myelin and IgMs for 2 hours. The cells were lysed and analyzed for internalized CNPase by capillary immunoblot. Full blots are provided in Supplementary Fig. 6d,e. (**d**) The line graph shows mean ± S.E.M. of CNPase/GAPDH ratio from 3 independent experiments. Statistical differences were calculated using 2-way ANOVA followed by Bonferroni posttests. Black asterisks indicate significant difference compared to Vehicle treated cells. Blue asterisks indicate significant difference compared to cells treated with isotype Ctrl IgM at the corresponding concentration. Red asterisks indicate significant difference compared to cells treated with rHIgM22 at the corresponding concentration (***p < 0.001; **p < 0.01; *p < 0.05).

Microglial cells can internalize extracellular material by pinocytosis, receptor-mediated endocytosis and antibody-mediated phagocytosis. All of these processes require cytoskeletal rearrangements and can therefore be inhibited with Cytochalasin D (CytoD), which acts by inhibiting actin polymerization^30^. To confirm that increases in pHrodo signal and intracellular CNPase indicate uptake of extracellular myelin, we pre-treated the BV-2 cells with CytoD for 30 minutes before addition of myelin and IgMs. Regardless of IgM treatment, pre-treatment with CytoD resulted in complete abolishment of myelin uptake under all conditions, as detected by pHrodo assay **(**Fig. 4a,b**)** and CNPase capillary immunodetection analysis **(**Fig. 4c,d), without demonstrating a toxic effect on the cells **(**Supplementary Fig. 4). These data also support the notion that the observed increases in CNPase can be attributed to phagocytic uptake of myelin by BV-2 cells, rather than adhesion of myelin to the cell surface.

### rHIgM22-mediated phagocytosis of myelin requires activity of the IgM Fc domain and Complement Receptor 3

**Figure 5:**
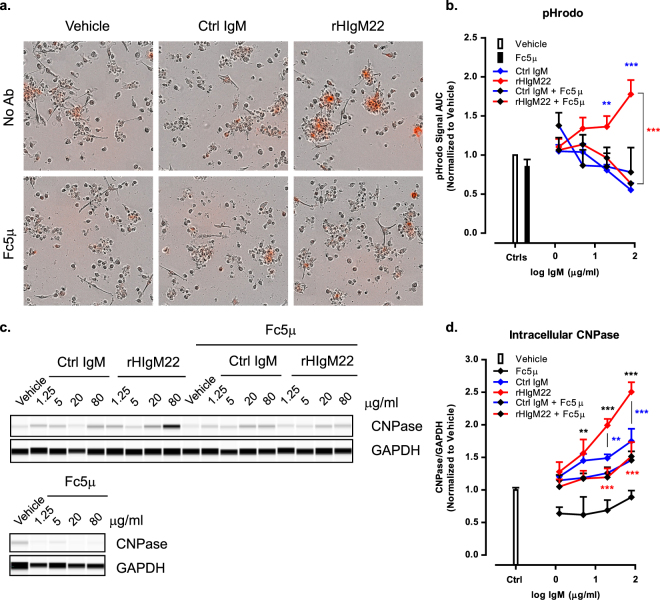
rHIgM22-mediated phagocytosis of myelin requires activity of the IgM Fc domain. (**a,b**) BV-2 cells were serum starved and IgMs were pre-incubated with Fc5µ antibody on ice for 30 minutes before treatment of cells with pHrodo-labeled myelin and IgMs. pHrodo signal was monitored on IncuCyte ZOOM for 24 hours. (**a**) Representative images of BV-2 cells treated with vehicle or IgMs (80 µg/mL) in the presence or absence of Fc5µ antibody at 24 hours after treatment. (**b**) Quantification of pHrodo uptake over 24 hours represented as area under the curve. The line graph shows the mean ± S.E.M. from 6 independent experiments. Statistical differences were calculated using 2-way ANOVA followed by Bonferroni posttests for rHIgM22 and isotype Ctrl IgM comparison (blue asterisks) and linear regression analysis for comparison to vehicle (details provided in Supplementary Fig. 5). (**c**) BV-2 cells were serum starved and IgMs were pre-incubated with Fc5µ antibody on ice for 30 minutes before treatment of cells with myelin and IgMs for 2 hours. The cells were lysed and analyzed for internalized CNPase by capillary immunoblot. Full blots are provided in Supplementary Fig. 6f,g and Supplementary Fig. 7a,b. (**d**) The line graph shows mean ± S.E.M. of CNPase/GAPDH ratio from 3 independent experiments. Statistical differences were calculated using 2-way ANOVA followed by Bonferroni post-tests. Black asterisks indicate significant difference compared to Vehicle treated cells. Blue asterisks indicate significant difference compared to cells treated with Isotype Ctrl at the corresponding concentration. Red asterisks indicate significant difference compared to cells treated with rHIgM22 at the corresponding concentration (***p < 0.001; **p < 0.01).

Our data suggest that general myelin internalization does not require presence of IgMs and myelin can be endocytosed at basal level in vehicle-treated cells. To specifically examine the potential role of IgM-mediated phagocytosis, we blocked the Fc region of IgMs, which is necessary for the antibody to be recognized by microglial cells. Pre-incubation of IgMs with the anti-Fc antibody (Fc5µ) specifically inhibited only the increase of pHrodo signal in response to rHIgM22 treatment **(**Fig. 5a,b**)**. Interestingly, addition of Fc5µ antibody resulted in a negative response to rHIgM22, comparable to that observed with the Ctrl IgM **(**Fig. 5b**)**. Similarly, capillary immunoblot analysis at 2 hours showed decreased levels of internalized CNPase in the presence rHIgM22 and Fc5µ compared to rHIgM22 treatment alone **(**Fig. 5c,d**)**. In both assays, pre-treatment with Fc5µ resulted in decreased phagocytosis only in rHIgM22-treated cells, without affecting responses to other treatments. These data indicate that the rHIgM22 Fc domain is required for augmentation of myelin phagocytosis by BV-2 cells.

**Figure 6:**
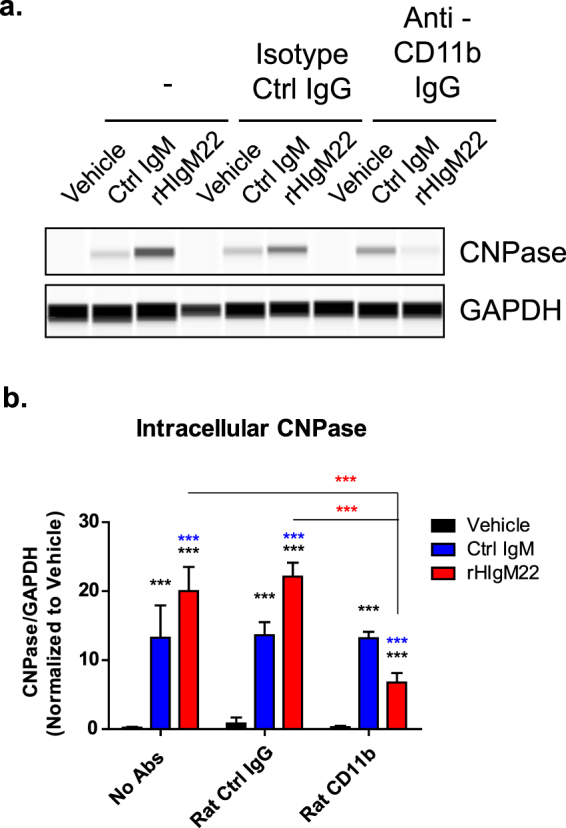
rHIgM22- mediated myelin phagocytosis requires activity of CR3. (**a,b**) BV-2 cells were serum starved and pre-incubated with an IgG antibody against CD11b (10 µg/mL) or an IgG isotype control antibody for 30 minutes, followed by treatment with myelin and IgMs (80 µg/mL) for 2 hours. The cells were lysed and analyzed for internalized CNPase by capillary immunodetection (**a**). Full blot is provided in Supplementary Fig. 7c. (**b**) The graph shows mean ± S.E.M. CNPase/GAPDH ratio from 3 independent experiments. Statistical differences were calculated using 2-way ANOVA followed by Bonferroni post-tests. Black asterisks indicate significant difference compared to Vehicle treated cells in the presence of corresponding IgG. Blue asterisks indicate significant difference compared to cells treated with isotype Ctrl IgM in the presence of corresponding IgG. Red asterisks indicate significant difference compared to cells treated with rHIgM22 across IgG pre-treatments (***p < 0.001).

The necessity of Fc function for rHIgM22-mediated myelin phagocytosis suggests that rHIgM22 may be acting through a receptor on BV-2 cells. Complement Receptor 3 (CR3) has recently been implicated in IgM-mediated phagocytosis^27^. CR3 consists of 2 subunits, CD11b and CD18^31^, and antibody-mediated blocking of CD11b inhibits CR3-mediated phagocytosis of IgM-opsonized material^27,31,32^. Therefore, we tested whether blocking CR3 activity with an anti-CD11b antibody could inhibit rHIgM22-mediated phagocytosis of myelin. In the absence of blocking antibodies, both IgMs resulted in enhanced levels of internalized myelin at 2 hours of treatment, with a higher level in rHIgM22-treated cells. Pre-treatment of cells with anti-CD11b antibody partially reduced levels of intracellular CNPase in rHIgM22-treated samples, without affecting responses to Ctrl IgM or vehicle **(**Fig. 6a,b**)**.

**Figure 7:**
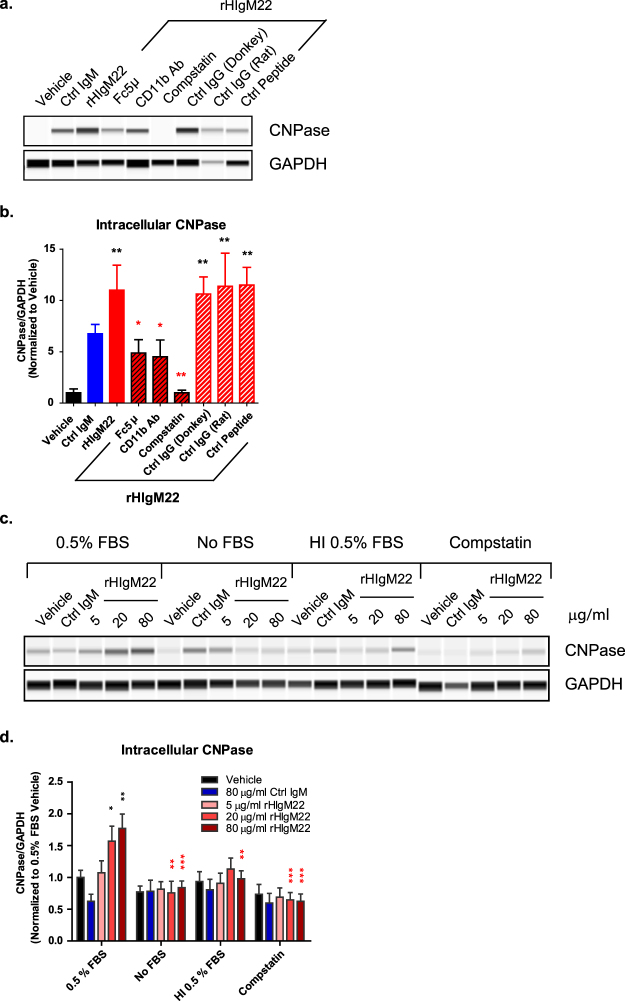
Activation of complement cascade is essential for rHIgM22-mediated myelin phagocytosis. (**a,b**) BV-2 cells were serum starved and pre-incubated with indicated treatments for 30 minutes prior to addition of rHIgM22 (80 µg/mL) and myelin. Each blocking IgG was used in conjunction with a host-matched isotype control IgG. A control peptide was used to verify specificity of complement pathway blocking effects of Compstatin. (**a**) At 2 hours after addition of myelin and indicated IgMs the cells were lysed and analyzed for internalized CNPase by capillary immunodetection. Full blots are provided in Supplementary Fig. 7d,e. (**b**) The graph shows the mean ± S.E.M. of CNPase/GAPDH ratio from 5 independent experiments. Statistical differences were calculated using 1-way ANOVA followed by Dunnett’s Multiple Comparison test. Black asterisks indicate significant difference compared to Vehicle treated cells. Red asterisks indicate significant difference compared to cells treated with rHIgM22 alone. (**c,d**) For serum comparison, BV-2 cells were serum starved in the presence or absence of FBS, HI FBS, and FBS with Compstatin. The cells were collected at 2 hours after treatment with myelin and IgMs and the cells were lysed and analyzed for internalized CNPase by capillary immunodetection. Full blots are provided in Supplementary Fig. 7f,g. (**d**) The graph shows mean ± S.E.M. CNPase/GAPDH ratio from 4 independent experiments. Statistical differences were calculated using 2-way ANOVA followed by Bonferroni post-tests. Black asterisks indicate significant difference compared to vehicle treated cells within the 0.5% FBS condition. Red asterisks indicate significant difference compared to cells treated with a corresponding concentration of rHIgM22 in the presence of 0.5% FBS (***p < 0.001; **p < 0.01; *p < 0.05).

Our findings show that rHIgM22-mediated myelin phagocytosis requires activation of a complement receptor. Therefore, we also tested the role of complement in our system. It is important to note that all assays were carried out in 0.5% serum media, which may have effectively served as a source of complement. To determine if complement activation is necessary for rHIgM22-mediated myelin phagocytosis, we pre-treated BV-2 cells with Compstatin (C3 inhibitor), prior to addition of myelin and IgMs, and analyzed cell lysates for internalized CNPase at 2 hours. While myelin uptake in cells pre-treated with Fc5µ or anti-CD11b antibody was partially inhibited **(**Figs 5 and 6**)**, pre-treatment with Compstatin completely blocked rHIgM22-mediated myelin uptake, reducing CNPase to the level detected in vehicle-treated cells **(**Fig. 7a,b). In addition, when BV-2 cells were serum starved and treated in the absence of FBS or presence of heat inactivated FBS, no effect of rHIgM22 treatment was observed **(**Fig. 7c,d). Together, these findings suggest that complement opsonization is essential, whereas multiple receptors may be involved.

## Discussion

While rHIgM22 has been shown to promote remyelination in animal models of MS^2,3^, much remains to be defined in terms of its cellular and molecular mechanisms of action. In order for remyelination to occur, OPCs need to be recruited to the lesion, where they need to proliferate, differentiate and finally remyelinate axons^33^. This implies that rHIgM22 would need to act at one of these stages of the OPC transition to mature OLs. However, numerous attempts have failed to show *direct* effects of rHIgM22 on OPC survival, proliferation or differentiation^5,34^. In contrast, treatment of mixed glial cultures, consisting of OPCs, astrocytes and microglia with rHIgM22 promotes proliferation of OPCs, suggesting that paracrine signaling may be an essential component of its function^5^.

The lack of observable effects in purified OPC cultures may be explained by absence of detectable binding of rHIgM22 to undifferentiated OPCs. In contrast, rHIgM22 shows robust binding to differentiated OLs and mature CNS myelin^2,7,35^. Since remyelination is heavily driven by differentiation of premature OPCs, rather than terminally differentiated OLs^33^, we decided to focus on the robust ability of rHIgM22 to bind CNS myelin that is produced by mature OLs. If rHIgM22 function begins with it binding to myelin-associated antigen, then studying this process should help identify the first responding cell type, which may then activate other glial cells through paracrine signaling.

A demyelinating event often results not only in loss of intact myelin sheaths, but also accumulation of myelin debris at the lesion site^24^. Microglia can recognize cellular debris, including damaged myelin, and remove it, allowing for a more pro-regenerative environment to be created in the CNS^20^. Microglial cells can ingest extracellular material by pinocytosis, receptor-mediated endocytosis and/or phagocytosis^18,36^, where the two latter processes can be augmented by the presence of an antigen-binding antibody. In this study, we have shown that rHIgM22 augments BV-2 microglial cell phagocytosis of myelin *in vitro*. This function of rHIgM22 is likely due to its ability to bind CNS myelin, as a non-specific Ctrl IgM did not augment myelin phagocytosis to the same extent. Moreover, cells treated with rHIgM22 showed sustained uptake of myelin, increasing accumulation of the lysosomal pHrodo signal over a 24-hour interval. In contrast, treatment with Ctrl IgM resulted in myelin uptake at early time points, but appeared to have a negative impact on long-term efficiency of myelin phagocytosis. At later time points, rHIgM22 treatment resulted in greater levels of internalized myelin compared to vehicle treatment, whereas treatment with Ctrl IgM appeared to lower basal levels of myelin uptake. The temporal dynamics of Ctrl IgM may be partially explained by initial non-specific binding to myelin, and/or related to differences in the microglial response to IgM that is mediated through activation of IgM receptors and sensitive to absence of cognate antigen.

In a similar study of antibody-mediated phagocytic clearance of extracellular α-synuclein by microglia, time course data indicated that treatment with an IgG antibody that binds α-synuclein accelerated phagocytosis of α-synuclein at early time points (up to 10 minutes)^21^. However, by 30 minutes after treatment, amounts of phagocytosed α-synuclein were similar in cells treated with anti-α-synuclein and control IgG antibodies. In contrast to these findings, internalized myelin levels were similar in rHIgM22 and Ctrl IgM-treated samples for 60 minutes following exposure of BV-2 cells, potentially pointing to a general IgM function in debris clearance that does not require interaction with antigen. However, only rHIgM22, which specifically binds myelin, promoted sustained myelin phagocytosis. The BV-2 pHrodo-based assays proved to be less sensitive than capillary immunodetection of CNPase, and did not show a difference in response to treatments for the first 6–8 hours.

In order to verify that increases in pHrodo signal and intracellular CNPase in response to rHIgM22 treatment can be attributed to phagocytic uptake of myelin, we demonstrated complete absence of both pHrodo and CNPase signal in the presence of CytoD, regardless of IgM treatment, indicating a requirement for intact cytoskeletal function for all forms of uptake of extracellular myelin.

To specifically target IgM-mediated phagocytosis without affecting other forms of cellular ingestion, we blocked the Fc domain of rHIgM22. As expected, pre-incubation of IgMs with the anti-Fc antibody specifically inhibited only the augmentation of myelin phagocytosis by rHIgM22, without affecting responses to treatment with Ctrl IgM. These data indicate that the Fc region of rHIgM22 is required for it to promote myelin phagocytosis by BV-2 cells. The necessity of Fc function also suggests that rHIgM22 may be acting through a receptor on BV-2 cells.

In contrast to IgG antibodies that have long been known to act through Fc gamma receptors, IgM antibody receptors are just beginning to be characterized. While IgM antibodies are classically thought to bind^37^ and signal through the Fcα/µ receptors^38^, complement receptors and scavenger receptors have been specifically implicated in IgM-mediated phagocytosis^39,40^. Furthermore, CR3 in particular has been shown to be the main receptor driving myelin phagocytosis^32^. More recently, Weinstein *et al*. showed that IgM-mediated microglial phagocytosis requires activity of CR3, rather than Fcα/µR^27^. In our system, blocking CR3 specifically inhibited rHIgM22-mediated phagocytosis, suggesting that myelin-bound rHIgM22 acts, at least in part, by activating CR3 in BV-2 cells. Since blocking CR3 did not affect myelin uptake in the vehicle or Ctrl IgM-treated cells, these results suggest that Ctrl IgM acts through an alternative mechanism that does not require CR3 activation. In addition, the finding that blocking CR3 did not reduce the phagocytic response in rHIgM22-treated cells to levels observed in vehicle-treated cells, suggests that multiple receptor-mediated pathways may be involved.

Throughout the study we consistently observed that the Ctrl IgM inhibits basal levels of myelin uptake over long periods of time, as observed in the pHrodo assays. Microglia express a large repertoire of receptors capable of binding antibodies, leading to a complex network of positive and negative signals that are associated with differential activity of immunoreceptor tyrosine-based activation and inhibition motifs (ITAM/ITIM)^41^. Therefore, one potential explanation of control IgM-mediated inhibition may be that Ctrl IgM may bind to an ITIM-associated receptor, leading to activation of an inhibitory signaling cascade. In addition, certain receptors, such as Fcα/µR, can activate or inhibit phagocytic signaling depending on the type of ligand bound^42–44^. The type of signaling appears to be primarily determined by whether or not the receptor-bound antibody carries an antigen. In presence of antigen, the immune complex induces crosslinking of receptors, leading to activation of signals resulting in phagocytosis. Binding of an antibody alone does not promote crosslinking, resulting in transduction of inhibitory signals, that are thought to dampen excessive immune responses^43^. It appears that treatment of microglia with Ctrl IgM results in the latter scenario, consistent with inability of Ctrl IgM to bind myelin. Conversely, other antibodies that bind CNS myelin (such as O4 antibody) are also able to promote remyelination^45^. Further studies are necessary to elucidate the differential signal transduction pathways activated by different IgMs.

In contrast to pHrodo data collected over 24 hours, biochemical analysis of myelin internalization at 2 hours after treatment does not demonstrate a negative response to Ctrl IgM. As CNPase uptake time course data suggests, the responses to different IgMs only begin to diverge at 2 hours after treatment. Therefore, the 2-hour time point of biochemical analysis may not capture inhibitory effects of Ctrl IgM, mediated via ITIM-type signal transduction pathways.

IgM antibodies can be directly recognized by certain receptors^37^ and induce phagocytosis of cellular debris in the absence of complement activation^15^. However, binding of complement factors can also act as direct “eat me” signals when bound to apoptotic cells and cellular debris, as well as amplify IgM-mediated phagocytosis of IgM-opsonized material^1^. C3 plays a central role in the complement pathway. When the complement cascade is activated, C3 becomes cleaved, resulting in deposition of C3b on the surface of target cells, which in turn acts as a ligand for complement receptors^46^. Myelin opsonization by complement has been shown to augment CR3-mediated myelin phagocytosis. The complement protein C3b, can directly opsonize myelin and present it as a ligand for CR3^39,47^. In addition to direct target opsonization, the cleavage of C3 and deposition of C3b can also result from IgM binding to antigen followed by complement activation. Furthermore, IgM-mediated phagocytosis has been shown to be significantly reduced in C3 null microglia^27^. Compstatin is a complement inhibitor that binds C3 and inhibits its proteolytic cleavage by C3 convertase^48^. Compstatin has previously been shown to inhibit monocytic phagocytosis of antibody-opsonized infected erythrocytes^49^. In our system, inhibition of C3 cleavage with Compstatin completely blocked rHIgM22-mediated phagocytosis of myelin, suggesting that complement activation is essential for this process. These findings were further supported by the abolishment of rHIgM22-mediated myelin phagocytosis in the presence of complement-inactivated serum and in the absence of serum, which serves as a source of complement components.

**Figure 8:**
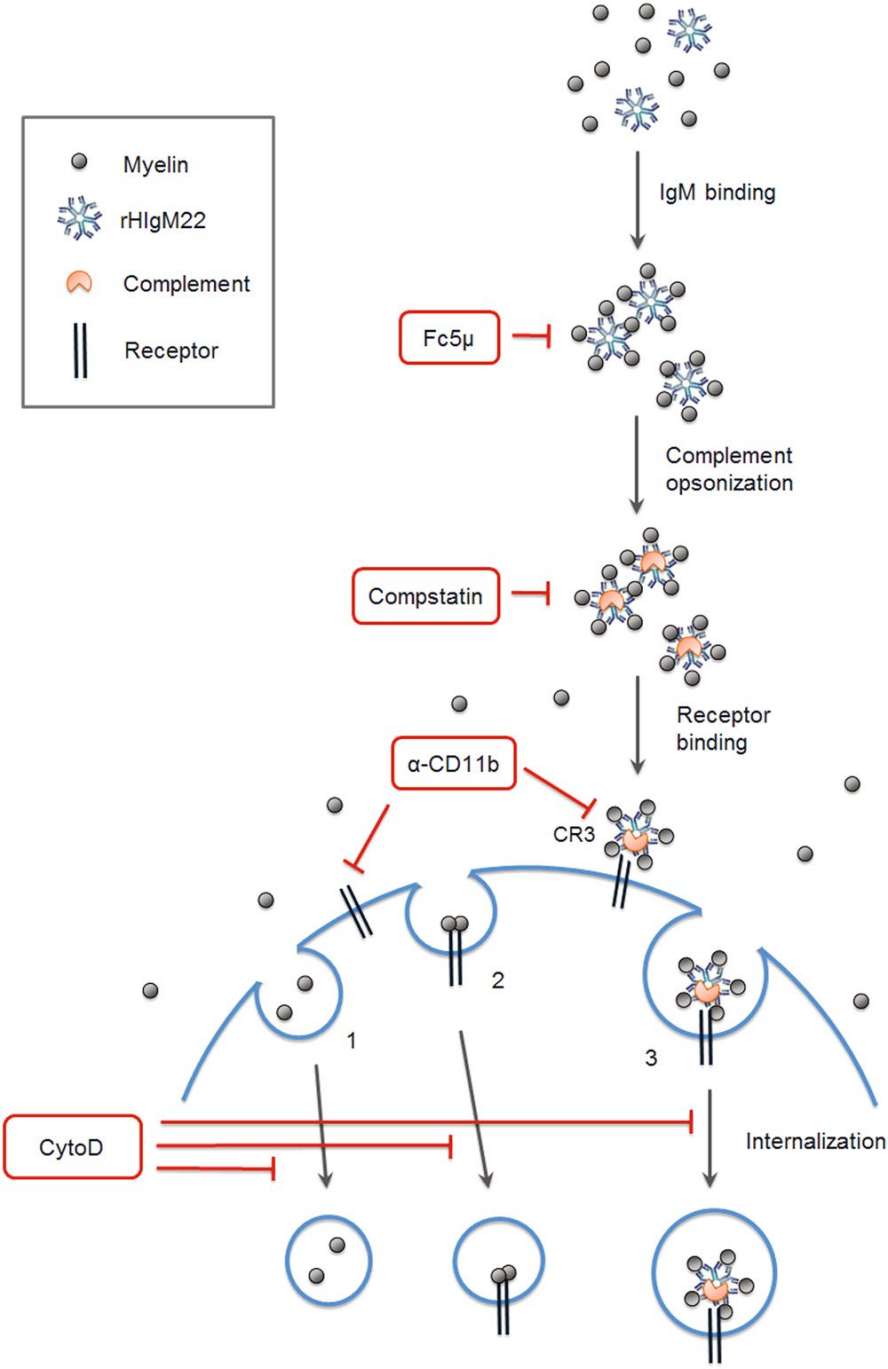
Proposed model for mechanism of rHIgM22-mediated myelin clearance. Schematic representation of potential pinocytosis (1), receptor-mediated endocytosis (2) and antibody-mediated phagocytosis (3) of myelin. rHIgM22 can bind and sequester exposed myelin debris, targeting it for phagocytosis. The Fc domain of rHIgM22 recruits complement components, leading to opsonization of the myelin/rHIgM22 complex. The complex is then internalized by microglial cells via the CR3 receptor.

Based on our findings and current literature, we propose the following model as one potential mechanism of action for rHIgM22 **(**Fig. 8**)**. In the context of damaged myelin debris, rHIgM22 binds and sequesters myelin, tagging it for phagocytic clearance by microglia. The rHIgM22/myelin complex is subjected to complement opsonization, making it a recognizable ligand for CR3 on microglial cells. Binding of rHIgM22/complement opsonized myelin to the receptor leads to phagocytic uptake of the complex, leading to clearance of extracellular myelin debris and disinhibition of OPC differentiation.

## Conclusions

rHIgM22 was discovered as a remyelinating antibody, but its molecular and cellular mechanisms of action remain incompletely defined. Early studies focused on potential activity of rHIgM22 as a signaling ligand that binds mature oligodendrocytes and elicits physiologic responses in target cells. However, direct effects on OPCs or oligodendrocytes were not readily detected, suggesting that paracrine mechanisms might be involved^5^. Here we show that microglia may play an important role in rHgIM22-induced remyelination by phagocytosing rHIgM22-bound myelin debris, thereby potentially disinhibiting OPC differentiation in demyelinated lesions of MS patients and possibly mediating further paracrine sequelae favorable for remyelination. Further studies will focus on how rHIgM22-mediated myelin phagocytosis may affect the microglial phenotype to promote a more regenerative environment for the remyelinating OPCs.

## Methods

### Myelin isolation and labeling

The myelin enriched fraction (MEF) was collected from adult rat brains and purified on a sucrose gradient, similar to a method described previously^50^. Briefly, whole rat brain homogenate was prepared by homogenizing frozen brain tissue in lysis buffer (1.0 M Sucrose, 150 mM NaCl, 10 mM TES, pH 7.5), followed by centrifugation at 85,000 × g for 28 minutes, to collect the myelin fraction. The myelin fraction was homogenized again and loaded on top of a linear sucrose gradient followed by centrifugation at 85,000 × g for 17 hours. The MEF was collected from between approximately 15–25% sucrose fractions, washed and resuspended in water. Myelin concentration was measured by using the BCA protein assay kit (ThermoFisher Scientific) and the presence of known myelin markers CNPase, MBP and MOG was verified by Western blot analysis. MEF was stored at −80 °C until further processing.

For myelin phagocytosis assays, individual aliquots of MEF were labeled with pHrodo red, succinimidyl ester (ThermoFisher Scientific), which can be used to detect lysosomal uptake of labeled proteins. pHrodo is a pH-sensitive dye, which is non-fluorescent at neutral pH, and fluoresces under acidic conditions. The pHrodo dye was dissolved in dimethyl sulfoxide (DMSO) and used at 1 µL/100 µg myelin. Myelin was resuspended in PBS and incubated with pHrodo for 45 minutes at room temperature, centrifuged and resuspended in PBS^29^. Further dilutions were made in cell culture medium just before application of myelin to cells.

### BV-2 cell culture and phagocytosis assays

The mouse microglial cell line (BV-2) was cultured in RPMI medium containing 10% FBS and 1% penicillin/streptomycin. For pHrodo assays, cells were cultured in 96-well plates at 25,000 cells/well, serum starved in DMEM with 0.5% FBS and 1% penicillin/streptomycin for 2 hours, and treated with 2.5 µg/mL pHrodo-labeled myelin and rHIgM22 or ChromPure Human IgM (Jackson ImmunoResearch), further referred to as Ctrl IgM. Triplicate wells were used for each treatment. Time-lapse videomicroscopy sequences of living BV-2 cultures were obtained using the IncuCyte ZOOM System (Essen BioScience), by acquisition of images from 4 fields per culture well every 2 hours for 24 hours. pHrodo red fluorescent signal was used as an indicator of myelin uptake by the lysosomal cell compartments^29^. Total integrated intensity of red fluorescence was calculated for each time point and plotted as a time course. Data were transformed into dose response curves by calculating the area under the curve (AUC) for pHrodo signal for each treatment. To control for myelin-specific responses, myelin was replaced with HEK293 cell membranes (PerkinElmer) in a parallel set of experiments.

For biochemical analysis of myelin phagocytosis, BV-2 cells were cultured in 6-well plates at 600,000 cells/well, serum starved for 2 hours, and treated with 2.5 µg/mL unlabeled myelin and IgMs (at indicated concentrations) for 2 hours. Cells were collected and lysed in RIPA buffer supplemented with protease and phosphatase inhibitor cocktails. After determination of protein concentration by BCA assay (ThermoFisher Scientific), the cell lysates were subjected to capillary electrophoresis immunoassay analysis (Simple Western) of CNPase (46/48 kDa, Millipore) and GAPDH (40 kDa, Abcam) using the WES™ instrument (ProteinSimple) following the manufacturer’s instructions. Briefly, cell lysates were mixed with a master mix (ProteinSimple) to give a final concentration of 0.2–0.4 mg/mL total protein. The samples were mixed with 1x sample buffer, 1x fluorescent molecular weight markers and 40 mM DTT and heated at 95 °C for 5 minutes. Through execution of a fully automated WES capillary system program separation and stacking matrices, assay samples, blocking solution, primary antibodies, horseradish peroxidase-conjugated secondary antibodies and chemiluminescent substrate were sequentially loaded and run in designated capillaries of a 25-sample cassette. The default WES program was used with the following modifications: stacking matrix load time was increased to 21.0 seconds, sample load time was set at 12.6 seconds and separation time was set for 31 minutes. The data were analyzed using Compass software (ProteinSimple) for peak areas of CNPase and GAPDH signal, and CNPase signal was normalized to GAPDH signal of same sample.

### Primary microglia

The work described below was approved by the Acorda Institutional Animal Care and Use Committee (IACUC) and was conducted in accordance with the Guide for the Care and Use of Laboratory Animals (2011) and the Animal Welfare Act. Timed pregnant Sprague Dawley rats, arriving at research site on E17, were obtained from Charles River Laboratories and singly housed in appropriately sized cages with enrichment. Rats had access to Harlan Teklad Global 14% Protein Rodent Maintenance diet and filtered water *ad libitum* throughout the study. Pups at age P2 were humanely euthanized by decapitation. Mixed glial cultures were prepared from P2 brain cortices and cultured on poly-D-lysine-coated flasks for 1 week. The cultures were then shaken at 200 RPM overnight, followed by replating of detached cells on bacteriological plates^51^. Non-adherent cells were washed away, and the adherent cells (purified microglia) were replated in 96-well plates. Myelin phagocytosis assays were carried out using the same protocol as for BV-2 cells, with the exception of a shorter serum starvation period of 1 hour.

### Inhibition of phagocytosis

To globally inhibit uptake of extracellular material, BV-2 cells were pre-treated with 1 µM Cytochalasin D (R&D Systems) for 30 minutes before addition of myelin and IgMs. To block activity of the Fc domain of IgMs, rHIgM22 and Ctrl IgM were pre-incubated with a donkey IgG Fc5µ antibody (Jackson ImmunoResearch) for 30 minutes on ice before addition to BV-2 cells. Fc5µ antibody was used at 1:2 mass ratio (µg) to rHIgM22 (~2.7:1 molar ratio). For blocking activity of CD11b component of CR3, BV-2 cells were pre-incubated with a rat IgG antibody against CD11b at ~2,400 IgG molecules/receptor (R&D Systems (M1/70)) (10 µg/mL) for 30 minutes before addition of myelin and IgMs. All blocking antibodies had matched isotype control antibodies from the corresponding vendors. For inhibition of complement pathway activation, BV-2 cells were pre-incubated with 12.5 µM Compstatin (R&D Systems) or control peptide for 30 minutes before addition of myelin and IgMs. For comparing responses under different serum conditions, the cells were serum starved for 2 hours in the presence of 0.5% FBS, no serum, or 0.5% heat inactivated (HI) FBS. HI FBS was prepared by incubating FBS at 65 °C for 30 minutes. The same serum conditions were kept for the duration of the experiment.

### Cytotoxicity assay

To determine if effects of Cytochalasin D (CytoD) may be attributed to cytotoxicity, BV-2 cells were treated with CytoD at a range of concentrations followed by addition of Cytotox Reagent (Essen BioScience). Cytotox Reagent is a highly sensitive cyanine nucleic acid dye that does not enter healthy cells with intact plasma membrane. In dying cells, plasma membrane integrity is compromised, allowing Cytotox to enter the cell and bind to DNA, resulting in a 100–1000-fold increase in fluorescent signal. Cytotox signal was tracked by time-lapse videomicroscopy sequences using the IncuCyte ZOOM System (Essen BioScience), by acquisition of images from 4 fields per culture well every 2 hours for 24 hours. Total Cytotox+ cell area was calculated for each time point and plotted as a time course. Data were transformed into a dose response curve by calculating the AUC for Cytotox signal for each treatment.

### Cell fractionation

BV-2 cells were serum starved, treated with IgMs (80 µg/mL) for 2 hours, and cells were fractionated using the Plasma Membrane Protein Extraction Kit (Abcam) according to manufacturer’s instructions. The fractions were analyzed for CNPase (Millipore), GAPDH (Abcam) and NaK-ATPase (Abcam) by SDS-PAGE immunoblot.

### Data analysis

All experiments were carried out at least three times (unless indicated otherwise), and the indicated values are represented as mean ± S.E.M. Statistically significant differences were determined by 2-way ANOVA followed by Bonferroni post hoc analysis. For pHrodo experiments where all responses were normalized to Vehicle, linear regression analysis was performed to compare responses to Ctrl IgM and rHIgM22 to Vehicle-treated cells. Significant responses were identified based on dose response curves that had slopes significantly different from zero. For all analyses significance was defined as p < 0.05.

### Ethics approval and consent to participate

All animal procedures required to obtain tissue for cell-based assays adhered to the Guide for the Care and Use of Laboratory Animals. These procedures were reviewed and approved by the Institutional Animal Care and Use Committee at Acorda Therapeutics, Inc.

### Availability of data and material

All data generated or analyzed during this study are included in this published article and its supplementary information files.

## Electronic supplementary material

Supplementary Figures and Tables
