# Supplementary material for: Human IgM antibody rHIgM22 promotes phagocytic clearance of myelin debris by microglia
Source: Sci Rep. 2018 Jun 20;8:9392. doi: 10.1038/s41598-018-27559-y (PMC6010437; doi:10.1038/s41598-018-27559-y)
Supplement: Supplementary file 1 — Supplementary Figures and Tables [file 41598_2018_27559_MOESM1_ESM.pdf]

Title page

Human IgM antibody rHIgM22 promotes phagocytic clearance of myelin debris by microglia

Authors:

Yana Zorina[1] – zorinay@mskcc.org

Jason Stricker[2] – jason.stricker@gmail.com

Anthony O. Caggiano[3] - tcaggiano@acorda.com

* Donald C. Button – donb@fastmail.com

1 Gene Editing and Screening Core Facility, Memorial Sloan Kettering Cancer Center, New York, NY, USA

2 Translational Medicine, Celgene Corporation, Summit, NJ, USA

3 Research and Development, Acorda Therapeutics Inc., Ardsley, NY, USA

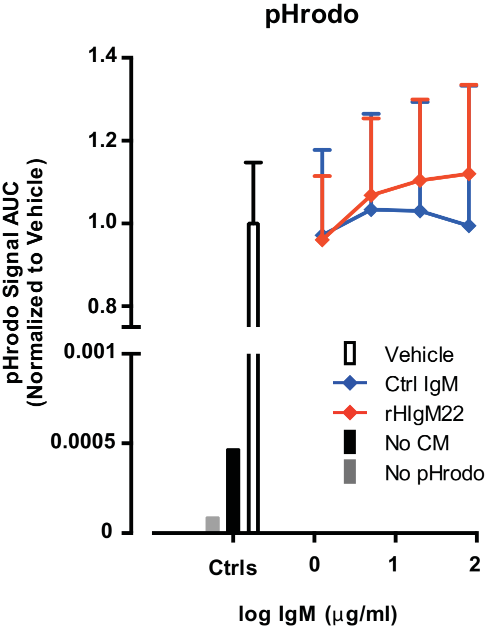

Supplementary Figure 1

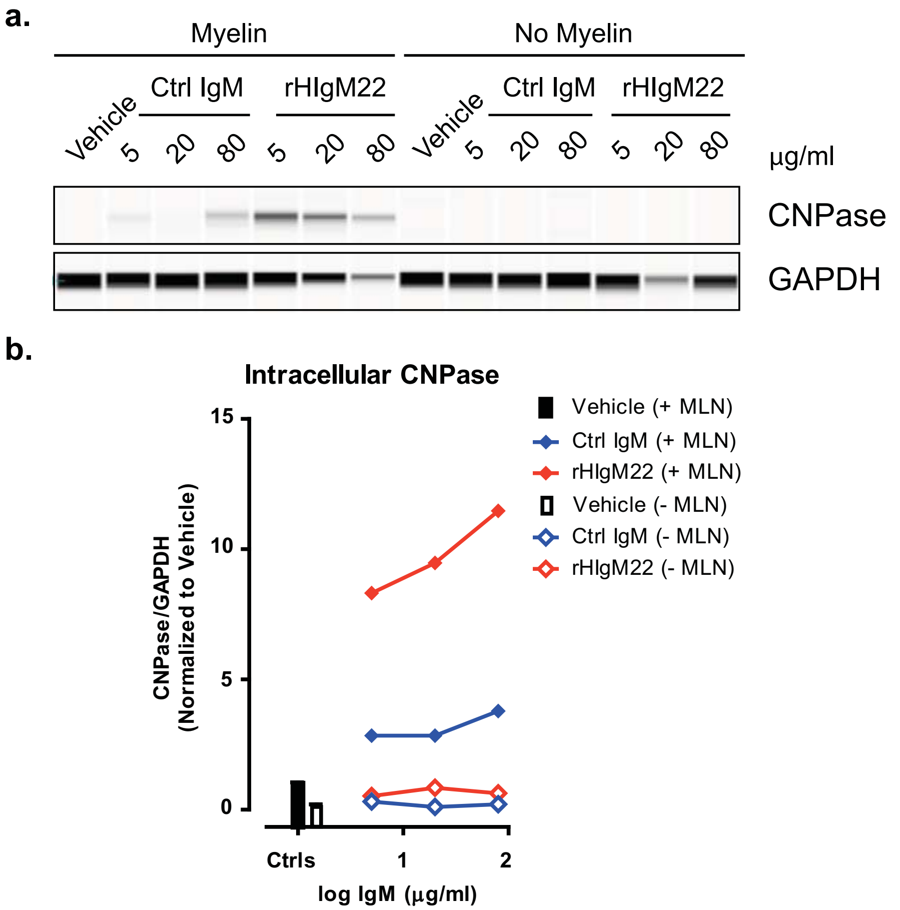

Supplementary Figure 2

a.

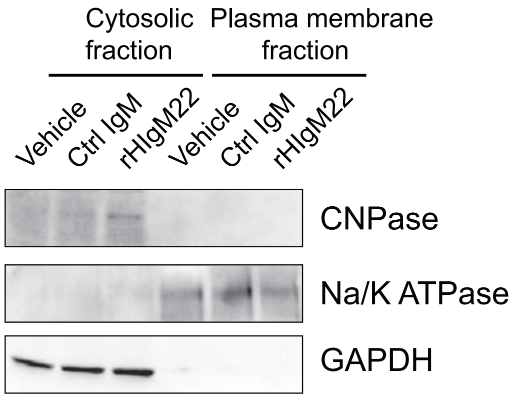

b.

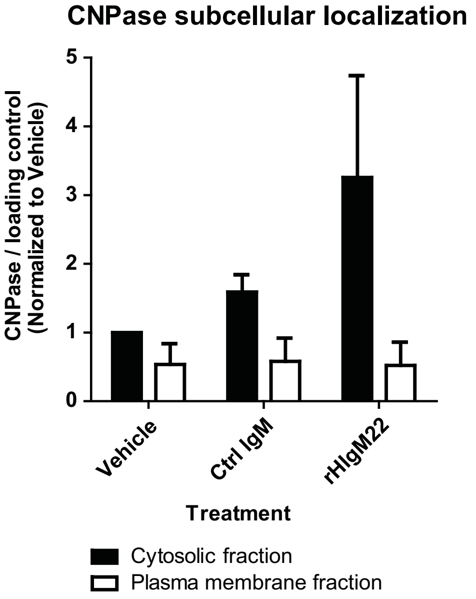

Supplementary Figure 3

a.

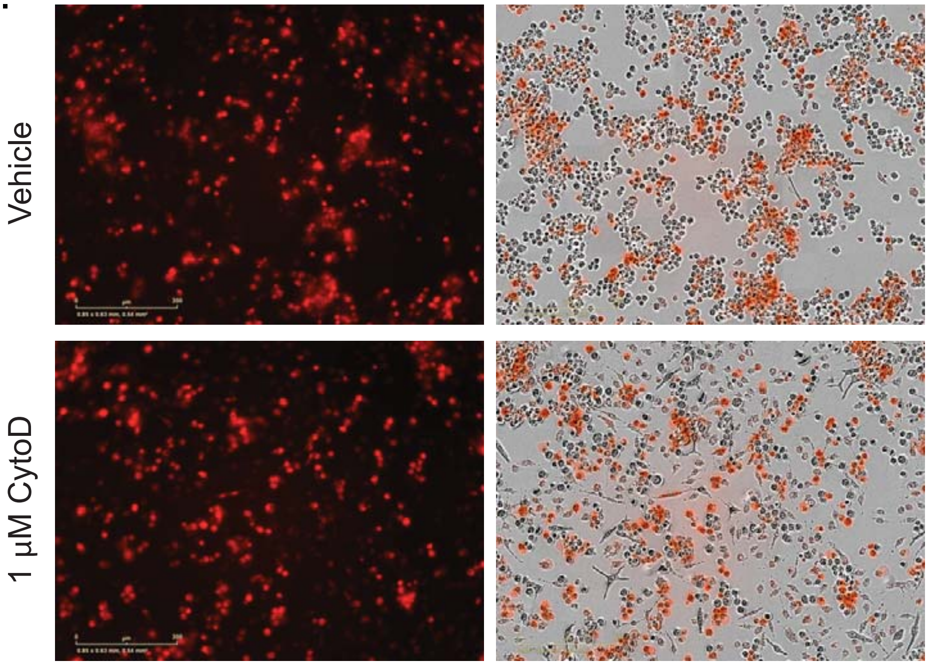

b.

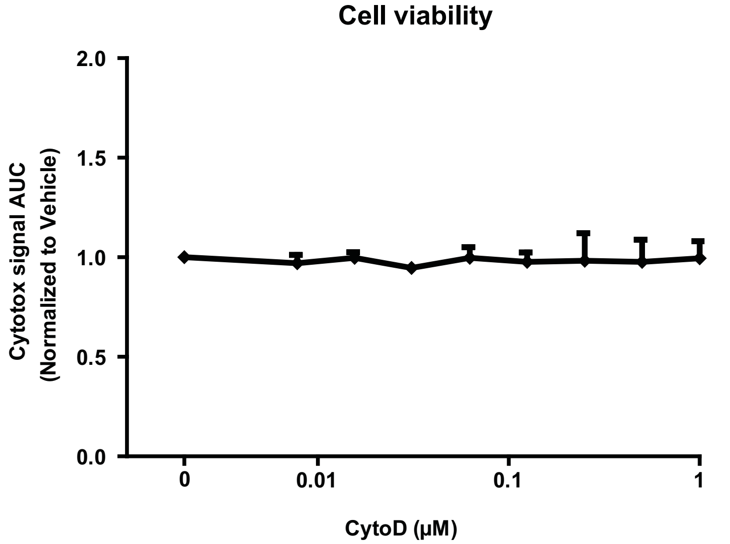

Supplementary Figure 4

a.

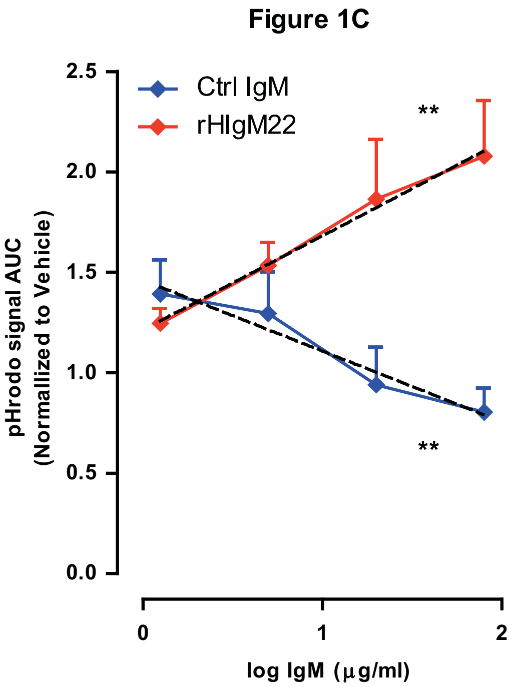

b.

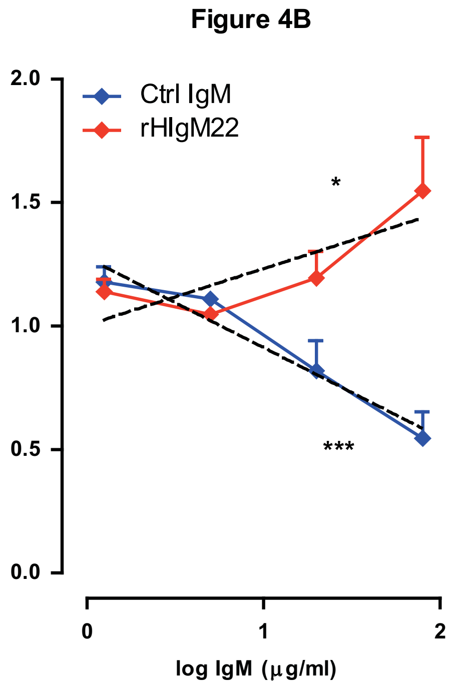

c.

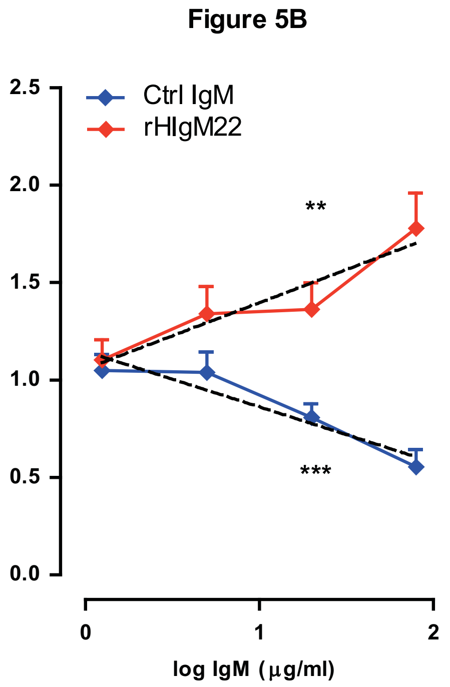

Figure 1C		
	Ctrl IgM	rHIgM22
r^2	0.2345	0.2662
Slope	-0.3525 ± 0.1249	0.4691 ± 0.1528
p-value	0.009	0.0049

Figure 4B		
	Ctrl IgM	rHIgM22
r^2	0.7639	0.3537
Slope	-0.3634 ± 0.06388	0.2282 ± 0.09755
p-value	0.0002	0.0414

Figure 5B		
	Ctrl IgM	rHIgM22
r^2	0.4606	0.325
Slope	-0.2848 ± 0.06569	0.3396 ± 0.1044
p-value	0.0003	0.0036

d.

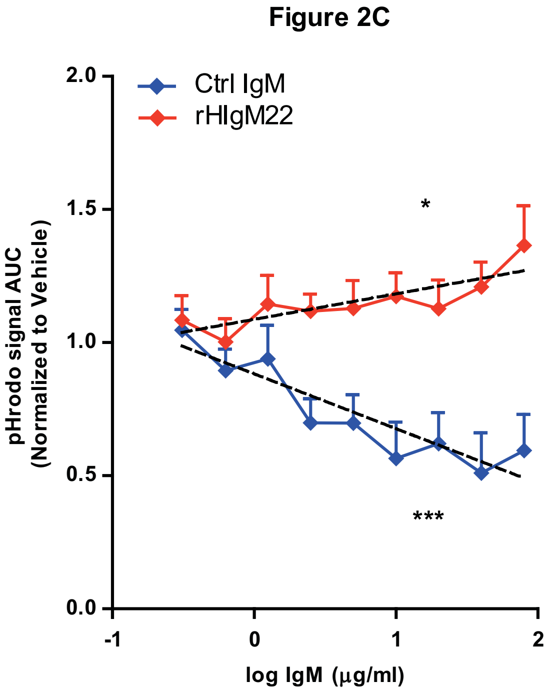

Figure 2C		
	Ctrl IgM	rHIgM22
r^2	0.2605	0.08452
Slope	-0.2057 ± 0.04762	0.09599 ± 0.04339
p-value	< 0.0001	0.0313

Supplementary Figure 5

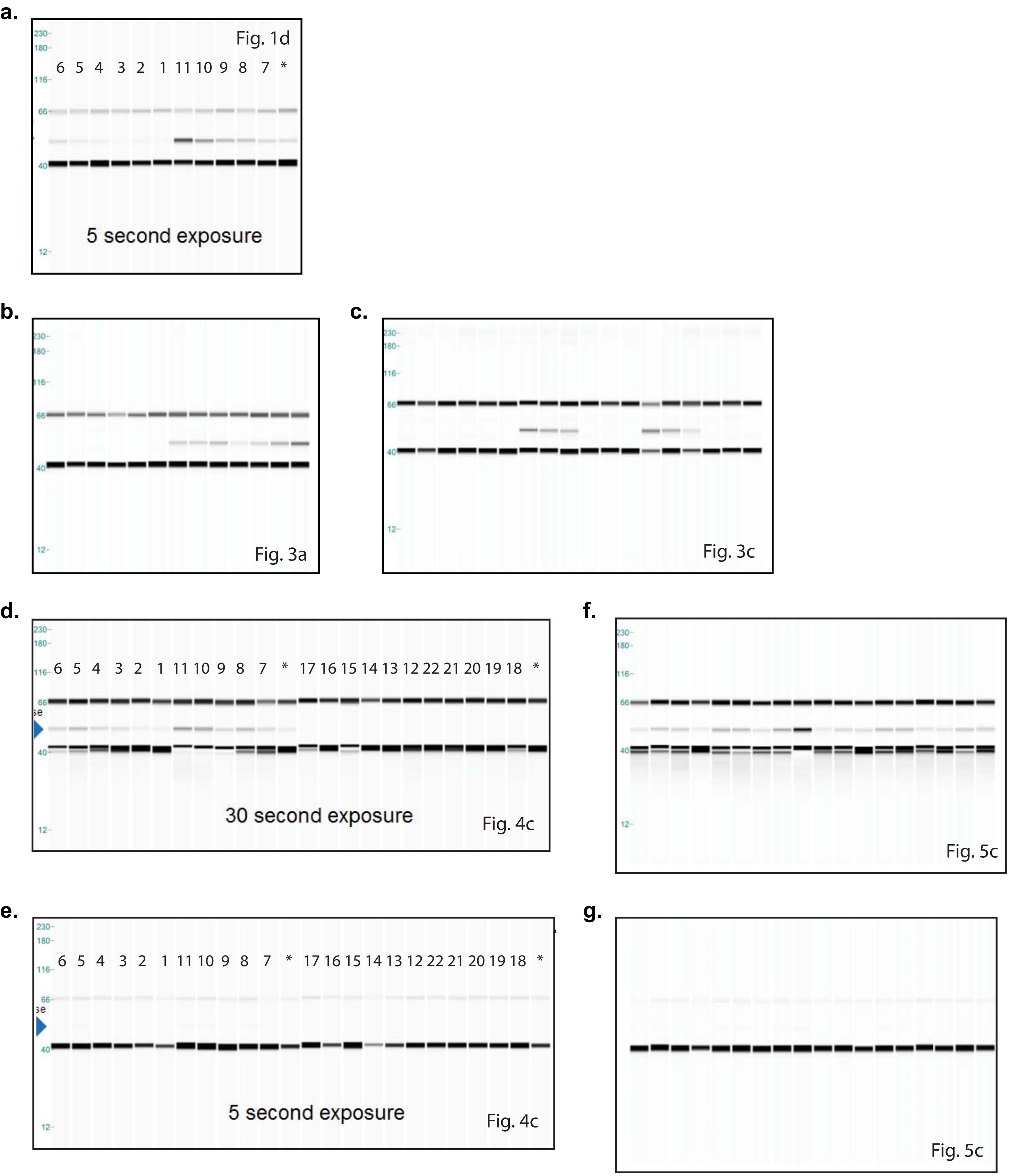

Supplementary Figure 6

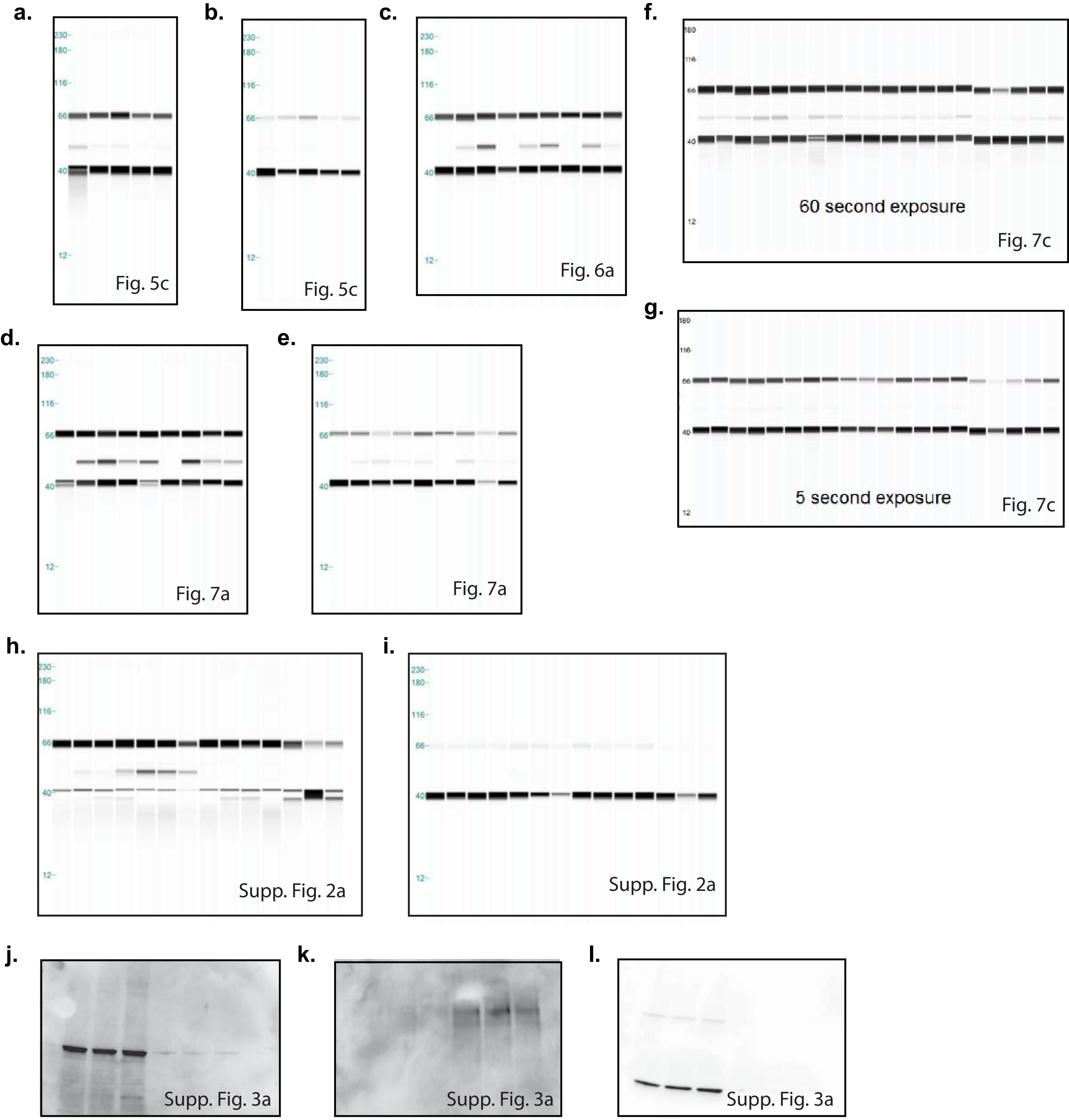

Supplementary Figure 7

Supplementary Figure 1: Stimulation of BV-2 cells with rHIgM22 has no effect on phagocytosis of HEK293 membranes. BV-2 cells were serum starved and treated with pHrodo-labeled HEK293 cell membranes and IgMs. pHrodo signal was monitored on IncuCyte ZOOM for 24 hours. pHrodo signal was measured over 24 hours and quantified as area under the curve. The line graph shows the mean ± S.E.M. from 4 independent experiments. 2-way ANOVA followed by Bonferroni posttests showed no difference in HEK293 membrane phagocytosis between rHIgM22 and Ctrl IgM treatment.

Supplementary Figure 2: CNPase is not synthesized in BV-2 cells in response to rHIgM22. BV-2 cells were serum starved and treated with IgMs in the presence or absence of myelin for 2 hours. The cells were lysed and analyzed for internalized CNPase by capillary immunodetection. The graph shows the CNPase/GAPDH ratio from a representative experiment.

Supplementary Figure 3: CNPase signal represents internalization, rather than adsorption of myelin. BV-2 cells were serum starved and treated with 80 µg/mL IgMs for 2 hours. The cells were fractionated to isolate cytosolic and plasma membrane fractions. (a) The fractions were run on SDS-PAGE and immunoblotted for CNPase. GAPDH and NaK ATPase were used as loading controls for cytosolic and plasma membrane fractions, respectively. (b) Levels of CNPase were normalized to the appropriate loading control of each fraction. The graph shows the mean ± S.E.M. of CNPase/Loading Control ratio from 4 independent experiments.

Supplementary Figure 4: Pre-treatment of cells with CytoD does not result in additional cell toxicity. (a) BV-2 cells were serum starved and treated with CytoD for 24 hours. Cytotox fluorescence signal was monitored on IncuCyte ZOOM for 24 hours. Representative images of

BV-2 cells treated with vehicle or CytoD (1 µM) at 24 hours after treatment are shown. (b) Quantification of Cytotox signal over 24 hours represented as area under the curve. The line graph shows the mean ± S.E.M. from 3 independent experiments.

Supplementary Figure 5: pHrodo dose response linear regression analysis. The graphs show pHrodo dose-response curves for Ctrl IgM and rHIgM22 treatments from Fig. 1c, 4b, 5b and 2c. The dotted black lines show linear regression fitted curves. The tables below each graph provide the details of linear regression analysis, including the r^2 value, line slope and p-value, comparing the slope to zero. The asterisks on the graphs indicate statistical difference of fitted line slope from zero, indicating a dose-response to IgM treatment (***, $p<0.001$; **, $p<0.01$; *, $p<0.05$).

Supplementary Figure 6: Full size capillary immunoblots for Fig. 1 and 3 - 5. All immunoblots show a non-specific band at ~66 kDa resulting from anti-CNPase antibody. The CNPase band is detected at ~46/48 kDa and GAPDH is detected at ~40 kDa. In some experiments the two bands could be detected at the same exposure, whereas in others a shorter time was required for GAPDH not to be overexposed. (a) Full size immunoblot for Fig. 1d. (b) Full size immunoblot for Fig. 3a. (c) Full size immunoblot for Fig. 3c. (d) Long exposure for CNPase in Fig. 4c. (a, d) Lanes were digitally rearranged in WES™ software (without changing any features other than order) for the main figures. The lane numbers indicate corresponding lanes in Fig. 1d and 4c. Asterisks indicate lanes that contained additional Vehicle controls that were not included in the main figures.

Supplementary Figure 7: Full size immunoblots for Fig. 5 – 7 and Supplementary Fig. 2 - 3. (a-i) Immunoblots show a non-specific band at ~66 kDa resulting from anti-CNPase antibody. The CNPase band is detected at ~46/48 kDa and GAPDH is detected at ~40 kDa.

The two bands were detected different exposure times in order for GAPDH not to be overexposed. (a) Long exposure for CNPase in Fig. 5c. (b) Short exposure for GAPDH in Fig. 5c. (c) Full size immunoblot for Fig. 6a. (d) Long exposure for CNPase in Fig. 7a. (e) Short exposure for GAPDH in Fig. 7a. (f) Long exposure for CNPase in Fig. 7c. (g) Short exposure for GAPDH in Fig. 7c. (h) Long exposure for CNPase in Supplementary Fig. 2a. (i) Short exposure for GAPDH in Supplementary Fig. 2a. (j - l) Traditional immunoblots for CNPase, Na/K ATPase and GAPDH in Supplementary Fig. 3a, respectively.

Supplementary Table 1: A single, representative pHrodo assay time course in BV-2 cells was analyzed by 2-way ANOVA followed by Bonferroni post-tests comparing each treatment to Vehicle. The table shows a comparison of each treatment to Vehicle at the corresponding time point (***, $p<0.001$; **, $p<0.01$; *, $p<0.05$).

Supplementary Table 2: A single, representative pHrodo assay time course in primary microglia was analyzed by 2-way ANOVA followed by Bonferroni post-tests comparing each treatment to Vehicle. The table shows a comparison of each treatment to Vehicle at the corresponding time point (***, $p<0.001$; **, $p<0.01$; *, $p<0.05$).

Supplementary Table 1:

BV-2 Myelin Phagocytosis Time Course 2-way ANOVA

	Isotype Ctrl IgM (µg/ml)				rHIgM22 (µg/ml)			
**Time (hrs)**	**1.25**	**5**	**20**	**80**	**1.25**	**5**	**20**	**80**
**0**	ns	ns	ns	ns	ns	ns	ns	ns
**2**	ns	ns	ns	ns	ns	ns	ns	ns
**4**	ns	ns	ns	ns	ns	ns	ns	ns
**6**	ns	ns	ns	ns	ns	ns	ns	ns
**8**	ns	ns	ns	***	ns	ns	ns	ns
**10**	ns	ns	***	***	ns	ns	***	ns
**12**	ns	*	***	***	ns	***	***	ns
**14**	ns	***	***	***	ns	***	***	***
**16**	ns	***	***	***	ns	***	***	***
**18**	ns	***	***	***	**	***	***	***
**20**	ns	***	***	***	***	***	***	***
**22**	ns	***	***	***	***	***	***	***
**24**	ns	***	***	***	***	***	***	***

Supplementary Table 2:

Primary Microglia Myelin Phagocytosis Time Course 2-way ANOVA

	Isotype Ctrl IgM (µg/ml)				rHIgM22 (µg/ml)			
**Time (hrs)**	**1.25**	**5**	**20**	**80**	**1.25**	**5**	**20**	**80**
**0.0000**	ns	ns	ns	ns	ns	ns	ns	ns
**2.000**	ns	***	***	***	ns	ns	ns	***
**4.000**	ns	***	***	***	ns	ns	ns	***
**6.000**	**	***	***	***	*	ns	ns	***
**8.000**	***	***	***	***	**	ns	ns	***
**10.00**	***	***	***	***	***	ns	ns	***
**12.00**	***	***	***	***	***	ns	ns	***
**14.00**	***	***	***	***	***	ns	ns	***
**16.00**	***	***	***	***	***	ns	ns	***
**18.00**	***	***	***	***	***	ns	ns	***
**20.00**	***	***	***	***	***	ns	ns	***
**22.00**	***	***	***	***	***	ns	*	***
**24.00**	***	***	***	***	***	ns	*	***
